# Quality of Life and Stress-Related Psychological Distress Among Patients with Cervical Cancer: A Cross-Sectional Analysis

**DOI:** 10.3390/diseases13030070

**Published:** 2025-02-25

**Authors:** Razvan Betea, Mirabela Dima, Veronica Daniela Chiriac

**Affiliations:** 1 Doctoral School, “Victor Babes” University of Medicine and Pharmacy Timisoara, 300041 Timisoara, Romania; razvan.betea@umft.ro; 2 Discipline of Neonatology, “Victor Babes” University of Medicine and Pharmacy Timisoara, 300041 Timisoara, Romania; 3 Department of Obstetrics and Gynecology, “Victor Babes” University of Medicine and Pharmacy Timisoara, 300041 Timisoara, Romania; chiriac.veronica@umft.ro

**Keywords:** cervical cancer, quality of life, stress, psychological, SF-36, WHOQOL-BREF, EORTC QLQ-C30, perceived stress scale

## Abstract

Background and Objectives: Cervical cancer places substantial burdens on physical and psychosocial health. This study evaluated changes in quality of life (QoL) and perceived stress in patients with cervical cancer before and six months after initiating standard treatment. Four validated instruments were used: the 36-Item Short Form Survey (SF-36), the Perceived Stress Scale (PSS), the World Health Organization Quality of Life-BREF (WHOQOL-BREF), and the European Organization for Research and Treatment of Cancer Quality of Life Questionnaire (EORTC QLQ-C30). Methods: Ninety-six women (mean age: 48.3 ± 7.4 years) with histologically confirmed cervical cancer were enrolled. Baseline (pre-treatment) assessments included SF-36, PSS, WHOQOL-BREF, and EORTC QLQ-C30. Follow-up was conducted six months after initiating either surgery or chemoradiation. Paired *t*-tests (or Wilcoxon signed-rank for non-parametric data) compared baseline and follow-up scores. Subgroup analyses were performed by disease stage (early vs. advanced) and marital status (married vs. unmarried). Results: Post-treatment assessments showed significant improvements in SF-36 physical functioning (from 61.9 ± 11.6 to 66.7 ± 12.3, *p* = 0.015) and mental health (from 63.4 ± 12.2 to 68.1 ± 12.4, *p* = 0.022). PSS scores declined from 23.2 ± 5.7 to 20.6 ± 5.5 (*p* = 0.001). WHOQOL-BREF physical and psychological domains increased, with physical health rising from 56.4 ± 12.0 to 60.7 ± 12.5 (*p* = 0.032). EORTC QLQ-C30 global health improved from 61.4 ± 13.8 to 66.3 ± 14.2 (*p* = 0.014). Advanced-stage patients exhibited greater absolute QoL gains yet remained below the early-stage scores. Married patients reported sharper reductions in PSS and higher final QoL scores. Conclusions: Over six months of standard cervical cancer treatment, these patients demonstrated significant QoL improvements and reduced perceived stress. While advanced-stage disease remained associated with lower post-treatment scores, these individuals benefited from notable gains compared to baseline. Marital status emerged as a protective factor. Ongoing psychosocial support may amplify these beneficial effects, warranting further longitudinal studies to optimize integrative survivorship care.

## 1. Introduction

Cervical cancer represents one of the most prevalent malignancies in women worldwide, with significant morbidity and mortality despite advances in screening and treatment [1,2]. Recent data indicate that timely diagnosis and intervention can prolong survival, yet many survivors grapple with ongoing physical and psychological sequelae [3]. While treatment modalities—ranging from surgery to chemoradiation—can effectively target the malignancy, these interventions often bring about side effects that impact patients’ day-to-day functioning [4]. Understanding these ramifications is crucial for delivering comprehensive care.

Quality of life (QoL) has become an increasingly important outcome measure in oncology research, reflecting a shift in focus from mere survival to overall well-being [5]. Patients with cervical cancer frequently encounter issues such as fatigue, pain, sexual dysfunction, anxiety, and depression, all of which significantly influence their QoL [6]. In clinical settings, validated instruments like the SF-36, WHOQOL-BREF, and EORTC QLQ-C30 help capture a multidimensional portrait of patients’ well-being.

However, the specific interplay between QoL and stress-related psychological distress in cervical cancer has not been fully elucidated. The Perceived Stress Scale (PSS) provides an additional layer of insight by quantifying the subjective experience of stress in the preceding weeks [7]. When combined with disease-specific QoL tools such as the EORTC QLQ-C30 and generic QoL measures like the SF-36 and WHOQOL-BREF, a holistic perspective on patient well-being emerges [8,9].

Cervical cancer disproportionately affects women in low- and middle-income regions, where social support systems might be fragile [10]. Furthermore, variations in marital status, educational level, and cultural norms can affect coping strategies and psychological resilience [11]. By investigating how these sociodemographic factors relate to QoL and psychological distress, clinicians can tailor supportive interventions to mitigate the burden of cervical cancer in diverse populations.

Though many studies focus on survival rates and tumor response, less attention is often paid to psychosocial outcomes within cervical cancer cohorts [12]. Given that chronic stress can exacerbate symptoms, compromise immune function, and potentially impact treatment adherence, a deeper exploration of stress and QoL is warranted [13]. This comprehensive approach may ultimately enhance clinical decision-making, improve patient satisfaction, and guide survivorship care models.

Therefore, the objectives of this cross-sectional study were twofold: (1) to evaluate QoL across multiple validated instruments in a cohort of women diagnosed with cervical cancer, and (2) to investigate perceived stress levels and their correlations with QoL outcomes. We also performed subgroup analyses by disease stage and marital status, hypothesizing that women with advanced-stage disease and those lacking social support might exhibit poorer QoL and higher stress.

## 2. Materials and Methods

### 2.1. Study Design and Ethical Considerations

We conducted a cross-sectional study of female patients diagnosed with cervical cancer between January 2021 and January 2023. All participants were recruited from the Timiş County Emergency Clinical Hospital “Pius Brinzeu”. Ethical approval was obtained from the institutional review board (IRB) of the hospital, and all procedures complied with the Declaration of Helsinki. Potential participants received an information sheet outlining the study objectives and then provided written informed consent.

Enrollment proceeded once eligibility was confirmed based on electronic medical records. Patients were not compensated, and participation was strictly voluntary. We emphasized that the study aimed to examine quality of life and psychological stress, with no impact on their standard clinical treatments. Data were collected through structured questionnaires and hospital medical charts and then stored in a password-protected database accessible only to the study team.

### 2.2. Participant Selection and Inclusion Criteria

In total, 120 patients with histologically confirmed cervical cancer were initially approached. The inclusion criteria were as follows: (1) age ≥18 years, (2) ability to comprehend and complete questionnaires in the local language, (3) no concurrent malignancies, and (4) undergoing either primary surgery or chemoradiation as per standard protocols. We excluded patients presenting with metastatic spread beyond the pelvis or other severe medical conditions (e.g., end-stage renal disease) likely to confound QoL assessments.

Of 120 patients, 15 declined to participate, and 9 returned incomplete questionnaires, yielding 96 participants (80% response rate) in the final analysis. Demographic factors (e.g., age, marital status), clinical data (e.g., FIGO stage, treatment type), and relevant comorbidities (e.g., diabetes, hypertension) were recorded. We classified disease into “early stage” (FIGO IA–IB2) and “advanced stage” (FIGO IIA–IVA) for subgroup comparisons [14].

### 2.3. Data Collection Instruments

All participants completed four standardized instruments. The SF-36 measures eight domains of health, summarized into Physical and Mental Component Scores (PCS, MCS). The WHOQOL-BREF evaluates physical, psychological, social, and environmental domains, using scaled scores (0–100). The EORTC QLQ-C30, specific to oncology, comprises functioning scales, symptom scales, and a global health scale [15]. Higher scores on functioning and global health reflect better QoL, while higher symptom scores indicate greater impairment. Psychological distress was assessed via the 10-item Perceived Stress Scale (PSS), which captures the degree to which individuals appraise their lives as unpredictable, uncontrollable, and overloaded. The PSS scores range from 0 to 40, with higher scores indicating greater perceived stress. Each participant completed the questionnaires independently, often in a dedicated quiet area of the outpatient clinic. A research coordinator was available to clarify any ambiguities.

### 2.4. Statistical Analysis

Data analysis was performed in SPSS version 27.0 (IBM Corp., Armonk, NY, USA). Continuous variables are presented as means with standard deviations (SD). Paired *t*-tests compared baseline vs. six-month follow-up scores for normally distributed data; Wilcoxon signed-rank tests were used otherwise. We calculated effect sizes (Cohen’s d) to gauge the magnitude of score changes.

Subgroup analyses (early vs. advanced stage; married vs. unmarried) employed repeated-measures ANOVA or Kruskal–Wallis tests when relevant, as well as independent *t*-tests for baseline differences. Correlations among changes in PSS and changes in QoL indices (ΔPSS vs. ΔSF-36 MCS, ΔWHOQOL-BREF Psychological, ΔEORTC QLQ-C30 Global) were examined using Pearson’s or Spearman’s rank correlation. Statistical significance was set at *p* < 0.05, two-tailed.

## 3. Results

Table 1 summarizes the demographic and clinical information for the 96 participants who completed both the baseline and six-month follow-up assessments. The average age at study entry was 48.3 (SD = 7.4) years, with a range from 32 to 68, indicating a midlife patient population. Two-thirds (66.7%) were married, while the remainder were unmarried (single, divorced, or widowed). Regarding disease severity, just under half (45.8%) had early-stage tumors (FIGO IA–IB2), whereas the majority (54.2%) were diagnosed at advanced stages (IIA–IVA). Treatment patterns reflected these clinical presentations, with 59.4% receiving chemoradiation (commonly favored in more advanced disease) and 40.6% undergoing primary surgery. Notably, about one-quarter of participants reported hypertension, and 16.7% had diabetes, underscoring the necessity of comprehensive management strategies that address comorbid conditions. Smoking habits were also recorded, revealing that 18.8% were current smokers, while 30.2% had a history of smoking.

Table 2 details changes in SF-36 domain scores between the baseline and six-month follow-up. Significant improvements emerged in several domains, notably Physical Functioning (*p* = 0.015), Bodily Pain (*p* = 0.039), General Health (*p* = 0.046), Vitality (*p* = 0.031), and Mental Health (*p* = 0.022). Effect sizes for these domains ranged from 0.29 to 0.41, suggesting small-to-moderate clinical impacts. The most pronounced gains were seen in Physical Functioning, which rose from 61.9 ± 11.6 to 66.7 ± 12.3, reflecting enhanced capacity for daily activities. While Role-Physical and Role-Emotional approached significance (*p* = 0.084, *p* = 0.052, respectively), the lack of definitive statistical change might reflect ongoing challenges in work-related or role-based tasks, possibly due to residual side effects or lingering emotional burdens. Social Functioning similarly trended upward but did not achieve the conventional threshold of significance (*p* = 0.068), indicating that many patients continued to experience social constraints, possibly from family responsibilities or limitations on leisure activities.

**Figure 1:**
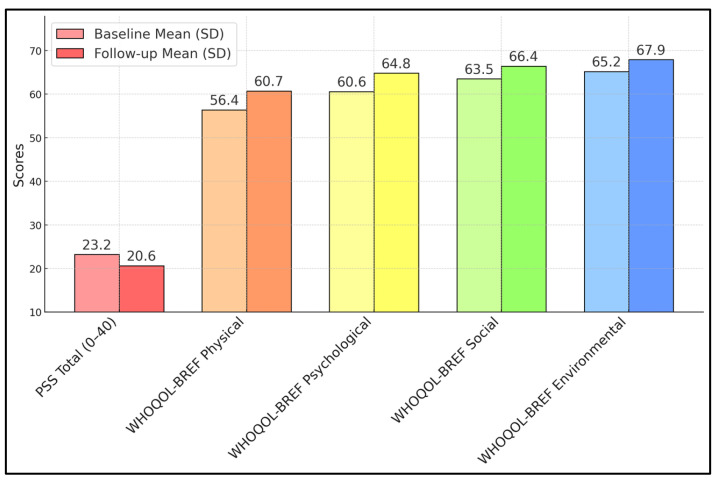
Changes in Perceived Stress Scale (PSS) and WHOQOL-BREF scores.

Table 3 highlights the evolution of the perceived stress and WHOQOL-BREF domains over six months of treatment. Notably, the PSS total score decreased significantly from 23.2 ± 5.7 to 20.6 ± 5.5 (*p* = 0.001), yielding an effect size of 0.46. This finding indicates a moderate reduction in the degree to which patients felt overwhelmed or stressed in their daily lives, perhaps reflecting adaptation to treatment regimens or psychological coping mechanisms becoming more effective over time. For the WHOQOL-BREF, improvements reached statistical significance in both the Physical and Psychological domains, with *p*-values of 0.032 and 0.040, respectively. The Physical domain’s increase from 56.4 ± 12.0 to 60.7 ± 12.5 aligns with the SF-36 data, suggesting better somatic well-being. Gains in the Psychological domain (60.6 ± 13.1 to 64.8 ± 13.4) mirror the decline in PSS, indicating an enhanced emotional outlook. Meanwhile, the Social and Environmental domains did trend upward but did not reach conventional significance (*p* = 0.089, *p* = 0.074), as seen in Figure 1.

Table 4 outlines changes in the EORTC QLQ-C30 scores, focusing on both functional and symptom scales. The Global Health scale exhibited a statistically significant increase from 61.4 ± 13.8 to 66.3 ± 14.2 (*p* = 0.014, d = 0.36), suggesting that patients generally perceived better overall well-being six months into treatment. Physical Functioning also improved significantly (*p* = 0.022), consistent with the SF-36 findings regarding enhanced capacity to engage in daily tasks. Emotional Functioning rose from 63.2 ± 14.2 to 67.1 ± 14.4 (*p* = 0.045), albeit with a smaller effect size, indicating that some emotional benefits accrued during this period. Symptom scales also shifted favorably. Fatigue dropped from 53.7 ± 16.4 to 48.2 ± 15.9 (*p* = 0.020), an effect that could reflect partial recovery from acute side effects like anemia, pain, and psychological distress. Pain scores similarly decreased (*p* = 0.037), aligning with improvements noted in the SF-36 Bodily Pain domain. Nausea and vomiting scores followed suit, declining significantly from 22.7 ± 11.3 to 19.4 ± 10.7 (*p* = 0.042).

**Figure 2:**
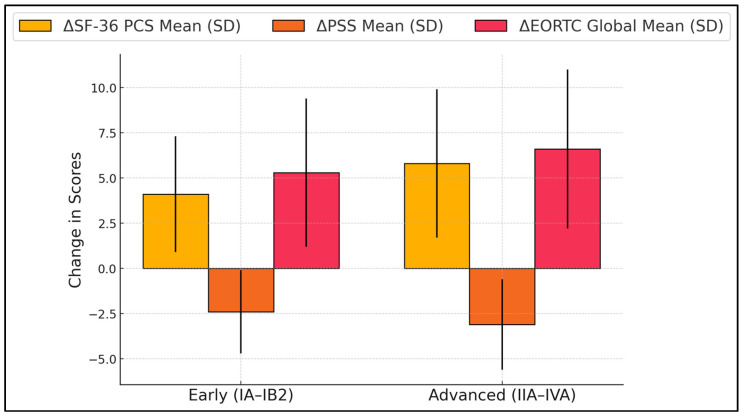
Subgroup analysis: early vs. advanced stage.

Table 5 compares the mean changes (Δ) in SF-36 Physical Component Summary (PCS), PSS scores, and EORTC QLQ-C30 Global Health between early-stage and advanced-stage patients. Interestingly, while advanced-stage patients had significantly lower baseline scores, their absolute improvements in these domains often outpaced those of early-stage counterparts. For instance, advanced-stage participants showed a +5.8 rise in PCS vs. +4.1 in early-stage (*p* = 0.042). This pattern may stem from a greater margin for improvement when starting from more compromised functioning. Both groups experienced meaningful drops in perceived stress, with advanced-stage patients reporting a mean ΔPSS of −3.1 compared to −2.4 in early-stage (*p* = 0.039). Similarly, EORTC Global Health scores increased by +6.6 in advanced-stage and +5.3 in early-stage (*p* = 0.021), suggesting that even those initially grappling with more severe disease can achieve substantial QoL gains when treatment is successful (Figure 2).

Table 6 presents the correlations among changes (Δ) from baseline to follow-up in perceived stress (PSS) and key mental health-related domains: SF-36 Mental Health, WHOQOL-BREF Psychological, and EORTC QLQ-C30 Global Health. All correlations are statistically significant at *p* < 0.01. Notably, ΔPSS shows a moderately strong negative relationship with ΔWHOQOL-BREF Psychological (r = −0.55) and ΔSF-36 Mental Health (r = −0.52), suggesting that individuals reporting a greater reduction in stress tended to exhibit more substantial improvements in psychological well-being and mental health. Additionally, changes in the WHOQOL-BREF Psychological domain correlated positively with changes in QLQ-C30 Global (r = 0.52), underscoring the broader interplay between emotional health and overall perception of wellness in a cancer context. Likewise, SF-36 Mental Health correlated moderately with EORTC QLQ-C30 Global Health (r = 0.45), further confirming that emotional gains and enhanced overall QoL are often linked.

## 4. Discussion

### 4.1. Literature Findings

This pre–post evaluation of women undergoing standard treatment for cervical cancer reveals notable improvements in multiple QoL dimensions, alongside a significant reduction in perceived stress over six months. Our findings align with previous research indicating that individuals often experience partial recovery from acute side effects (e.g., pain, fatigue) once they adapt to treatment regimens [12,13]. In particular, SF-36 domains related to physical functioning and mental health, as well as the WHOQOL-BREF physical and psychological domains, showed meaningful gains. Concomitantly, EORTC QLQ-C30 reflected a decline in common symptoms such as fatigue, pain, and nausea/vomiting, suggesting that symptom management strategies may be successful in improving daily functioning and global health perceptions.

Notably, patients with advanced-stage disease demonstrated higher absolute score increments than early-stage counterparts, albeit starting from more compromised baselines. This finding supports the notion that, despite the greater burden of disease and potentially more intensive therapy, advanced-stage patients can still realize substantial QoL and stress-related benefits [14,15,16]. Meanwhile, our correlation analyses underscore the tight linkage between psychological stress relief and overall well-being. Reduced PSS was consistently associated with boosts in mental health, psychological QoL, and global perceptions of health, highlighting the role of psychosocial factors in modulating cancer-related outcomes.

In examining the quality of life in cervical cancer patients, Yao Xie et al. [17] reported that patients experienced the lowest QOL scores one month after treatment, particularly those with early and advanced cancers, though scores gradually improved over the following months. In contrast, J Khalil et al. [18] found that long-term cervical cancer survivors up to ten years post-diagnosis generally maintained good overall QOL comparable to healthy controls but faced significant challenges in emotional and sexual functioning. Notably, Khalil et al. [18] highlighted that spiritual well-being and social support were crucial in positively influencing QOL, accounting for 81% of the variance in scores.

Prasongvej et al. [19] conducted their study among 192 women, including 97 cervical cancer survivors and 95 healthy controls, finding notable differences in QoL domains such as physical, role, emotional, and social functions. Specifically, survivors treated with radical hysterectomy reported higher scores in emotional and social functioning than the control group, indicating a better overall QoL. Conversely, those undergoing concurrent chemoradiation reported greater pain than controls. In contrast, Zeng et al. [20] reviewed various QoL instruments, with reported internal consistency ranging from 0.68 to 0.99 and test–retest reliability from 0.60 to 0.95, emphasizing the robustness and reliability of these tools in capturing the multifaceted impacts of cervical cancer on survivors’ lives.

Korfage et al. [21] evaluated HRQoL in a cohort of 291 Dutch women, 2–10 years post-diagnosis, utilizing tools like the SF-36 and EQ-5D. They found that, while overall generic HRQoL scores aligned closely with those of the general population, specific issues such as mental health, sexual worry, and treatment-related side effects like urinary leakage (15%) and crampy abdominal pain (17%) were more prevalent among survivors, especially those treated with primary radiotherapy. Similarly, Osann et al. [22] explored factors associated with poor QoL among 204 cervical cancer survivors in California, identifying that radiation treatment, combined with pre-existing comorbidities, significantly correlated with reduced QoL. They noted particularly high levels of depression and anxiety, with 63% of those in the lowest QoL quartile showing depression levels more than one standard deviation above the mean.

The studies by Lucia Membrilla-Beltran et al. [23] and David H. Mvunta et al. [24] explored the impact of cervical cancer treatments on the quality of life and sexuality of survivors, revealing significant challenges despite clinical success in treatment. Membrilla-Beltran et al. [23], through their retrospective case-control study involving 66 Spanish cervical cancer survivors, found substantial impairment in sexual function and satisfaction, with survivors experiencing dysfunction in almost half of the assessed domains. They reported frequent symptoms of pain and fatigue, highlighting a decreased QoL compared to healthy controls. In a similar manner, the study by Mvunta et al. [24] conducted at the Ocean Road Cancer Institute in Tanzania assessed QoL among 323 cervical cancer patients post-chemoradiotherapy. Their findings indicated that more than half of the patients reported a good overall QoL, but it was significantly influenced by factors such as education, smoking status, sexual partners, treatment modality, and time since treatment completion. Notably, patients who underwent combined external beam radiation and brachytherapy showed higher functioning across most QoL domains.

In this context, it is worth mentioning marital status effects and differential QoL changes. In a similar manner, the study by Qing Chen et al. [25] investigated the impact of marital status on the survival outcomes of cervical cancer patients using data from the Surveillance, Epidemiology, and End Results (SEER) database spanning from 1975 to 2017. The research found that married patients demonstrated significantly better overall survival (OS) and cervical cancer-specific survival (CCSS) compared to their unmarried counterparts, with a notable hazard ratio (HR) for OS at 0.830 (95% CI: 0.798–0.862) and for CCSS at 0.892 (95% CI: 0.850–0.937). Furthermore, the reduction in death risk for married patients was significant, with an HR of 0.723 for CCSS after accounting for competing risks, emphasizing the protective effect of marital status across the different time periods analyzed.

Similarly, Di Zhou and colleagues [26] focused on the prognostic influence of marital status specifically in cervical adenocarcinoma (AC), also using SEER data but for the years 2004 to 2015. This study confirmed that marital status is a beneficial factor, with married patients not only having better cancer-specific survival (CSS) and overall survival (OS) rates—80.16% and 78.26%, respectively, compared to 68.58% and 64.62% in unmarried patients—but also revealing that marital status remained an independent prognostic factor after multivariate analysis (CSS HR: 0.770; OS HR: 0.751). Both studies underscore the significant association between marital status and improved survival outcomes in cervical cancer patients, suggesting a possible psychosocial support mechanism that enhances the efficacy of treatment and management of the disease.

The clinical implications of the findings indicate that standard treatments for cervical cancer effectively improve both physical and psychological health outcomes. Significant improvements in quality of life for advanced-stage patients highlight the need for customized psychosocial interventions. Additionally, the beneficial impact of marital status on treatment outcomes emphasizes the importance of integrating social support mechanisms into treatment protocols. These insights suggest that a holistic treatment approach, including robust psychosocial support, is crucial for enhancing overall patient well-being and optimizing survivorship care in cervical cancer management.

Collectively, these results advocate for integrative care approaches that address not only the tumor but also the physical, emotional, and social ramifications of cervical cancer. While many domains improved over six months, areas like role functioning, social participation, and environmental stressors did not consistently reach statistical significance, suggesting ongoing challenges. Interventions such as psychosocial counseling, relaxation training, or peer support may further mitigate stress and enhance quality of life. Future research should incorporate longer follow-up to capture post-treatment survivorship trajectories and investigate whether these initial gains endure. Additionally, exploring the impact of marital status, educational background, and economic resources will help refine patient-centered interventions that recognize the diverse realities confronted by women with cervical cancer.

### 4.2. Study Limitations and Future Perspectives

While the cross-sectional design provided valuable insights into the QoL impacts at two specific time points—before treatment and six months post-treatment—this design inherently limits the ability to establish causality between cervical cancer treatments and changes in QoL or stress levels. Additionally, the reliance on self-reported measures might introduce bias, as participants’ responses could be influenced by their current state of mind or recall biases. The study sample, though adequate, represents a specific regional population, which may not generalize to other cultural or geographic contexts, particularly given the unique socioeconomic and healthcare dynamics in different regions. Furthermore, the study did not account for potential confounders such as participants’ psychological history or individual coping mechanisms, which could significantly affect the perceived QoL and stress levels. These factors need to be considered when interpreting the findings and in the planning of future research to better understand the complex dynamics between cervical cancer, treatment modalities, and long-term patient outcomes.

Future studies should consider a longitudinal design and the inclusion of a control group to better establish causal relationships and reduce potential biases from self-reported data. Additionally, exploring the influence of psychological history and coping strategies on quality of life outcomes could provide a more comprehensive understanding of the factors impacting cervical cancer patients.

## 5. Conclusions

Our longitudinal study highlights that patients with cervical cancer can achieve noteworthy QoL improvements and stress reduction within six months of commencing standard therapy. Physical functioning, bodily pain, vitality, and mental health all showed statistically significant enhancements on the SF-36, paralleling positive changes in the WHOQOL-BREF physical and psychological domains. Likewise, the EORTC QLQ-C30 confirmed better global health, reduced fatigue, and less pain by the six-month mark. Importantly, individuals with more advanced disease exhibited larger net gains despite presenting with more severe baseline deficits, underscoring the potential for substantial psychosocial recovery regardless of initial severity. Reductions in perceived stress correlated closely with improvements in multiple QoL measures, suggesting that comprehensive care should emphasize both medical and psychological support. These findings point to a robust capacity for functional and emotional recovery, although certain domains—particularly role-based tasks and social engagement—may require prolonged or specialized interventions. By integrating psychosocial services, stress management programs, and vigilant symptom monitoring into cancer care plans, clinicians may optimize well-being for women confronting cervical cancer. Further studies should assess long-term trajectories, aiming to sustain or enhance these initial benefits throughout survivorship.

## Figures and Tables

**Table 1:** Demographic and clinical characteristics of the final cohort.

Variable	Value
Age (years, mean ± SD)	48.3 ± 7.4 (range: 32–68)
Marital Status	Married: 64 (66.7%)
	Unmarried: 32 (33.3%)
Stage Classification	Early (IA–IB2): 44 (45.8%)
	Advanced (IIA–IVA): 52 (54.2%)
Primary Treatment	Surgery: 39 (40.6%)
	Chemoradiation: 57 (59.4%)
Comorbidities	Diabetes: 16 (16.7%)
	Hypertension: 24 (25.0%)
	Other: 11 (11.5%)
Smoking Status	Current: 18 (18.8%)
	Former: 29 (30.2%)
	Nonsmoker: 49 (51.0%)

SD—standard deviation.

**Table 2:** SF-36 scores at baseline vs. six-month follow-up.

Domain	Baseline Mean (SD)	Follow-Up Mean (SD)	*p*-Value	Effect Size (Cohen’s d)
Physical Functioning	61.9 (11.6)	66.7 (12.3)	0.015	0.41
Role-Physical	59.2 (13.1)	62.9 (13.4)	0.084	–
Bodily Pain	62.7 (11.8)	66.2 (12.6)	0.039	0.29
General Health	57.3 (10.7)	60.5 (11.3)	0.046	0.29
Vitality	60.4 (12.7)	64.6 (13.1)	0.031	0.33
Social Functioning	66.8 (10.9)	69.8 (11.5)	0.068	–
Role-Emotional	61.5 (12.4)	64.7 (12.9)	0.052	–
Mental Health	63.4 (12.2)	68.1 (12.4)	0.022	0.38

SF—short form; SD—standard deviation.

**Table 3:** Changes in Perceived Stress Scale (PSS) and WHOQOL-BREF scores.

Measure	Baseline Mean (SD)	Follow-Up Mean (SD)	*p*-Value	Effect Size (Cohen’s d)
PSS Total (0–40)	23.2 (5.7)	20.6 (5.5)	0.001	0.46
WHOQOL-BREF Physical	56.4 (12.0)	60.7 (12.5)	0.032	0.35
WHOQOL-BREF Psychological	60.6 (13.1)	64.8 (13.4)	0.04	0.32
WHOQOL-BREF Social	63.5 (14.5)	66.4 (15.1)	0.089	–
WHOQOL-BREF Environmental	65.2 (13.2)	67.9 (13.7)	0.074	–

SD—standard deviation.

**Table 4:** EORTC QLQ-C30: baseline vs. follow-up.

Scale/Domain	Baseline Mean (SD)	Follow-Up Mean (SD)	*p*-Value	Effect Size (Cohen’s d)
Global Health	61.4 (13.8)	66.3 (14.2)	0.014	0.36
Physical Functioning	63.7 (13.5)	68.4 (13.7)	0.022	0.34
Role Functioning	58.6 (14.1)	62.8 (14.8)	0.051	–
Emotional Functioning	63.2 (14.2)	67.1 (14.4)	0.045	0.27
Cognitive Functioning	65.3 (13.2)	68.8 (12.9)	0.058	–
Social Functioning	60.9 (15.0)	64.2 (15.8)	0.092	–
Symptom Scales				
Fatigue	53.7 (16.4)	48.2 (15.9)	0.02	0.34
Pain	46.8 (15.6)	42.1 (14.9)	0.037	0.31
Nausea/Vomiting	22.7 (11.3)	19.4 (10.7)	0.042	0.3

SD—standard deviation.

**Table 5:** Subgroup analysis: early vs. advanced Stage.

Stage	n	ΔSF-36 PCS Mean (SD)	ΔPSS Mean (SD)	ΔEORTC Global Mean (SD)	*p*-Values (PCS/PSS/EORTC)
Early (IA–IB2)	44	+4.1 (3.2)	−2.4 (2.3)	+5.3 (4.1)	0.042/0.039/0.021
Advanced (IIA–IVA)	52	+5.8 (4.1)	−3.1 (2.5)	+6.6 (4.4)	0.3

SF—short form; SD—standard deviation.

**Table 6:** Correlations among changes (Δ) in PSS, SF-36 Mental Health, WHOQOL-BREF Psychological, and EORTC QLQ-C30 Global.

Variable	ΔPSS	ΔSF-36 Mental Health	ΔWHOQOL-BREF Psych.	ΔQLQ-C30 Global	Effect Size (Cohen’s d) (PCS/PSS/EORTC)
ΔPSS	1	–0.52 **	–0.55 **	–0.42 **	
ΔSF-36 Mental Health	–0.52 **	1	0.48 **	0.45 **	-
ΔWHOQOL-BREF Psychological	–0.55 **	0.48 **	1	0.52 **	
ΔQLQ-C30 Global	–0.42 **	0.45 **	0.52 **	1	0.41/0.33/0.36

SF—short form; ** Spearman’s or Pearson’s coefficients; *p* < 0.01.

## Data Availability

Data available on request.
